# Extrusion 3D Printing of Paracetamol Tablets from a Single Formulation with Tunable Release Profiles Through Control of Tablet Geometry

**DOI:** 10.1208/s12249-018-1107-z

**Published:** 2018-08-10

**Authors:** Shaban A. Khaled, Morgan R. Alexander, Derek J. Irvine, Ricky D. Wildman, Martin J. Wallace, Sonja Sharpe, Jae Yoo, Clive J. Roberts

**Affiliations:** 1 Advanced Materials and Healthcare Technologies, School of Pharmacy, University of Nottingham, University Park, Nottingham, NG7 2RD UK; 2 Faculty of Engineering, University of Nottingham, University Park, Nottingham, NG7 2RD UK; 3 Advanced Manufacturing Technology, GlaxoSmithKline (Ireland), 12 Riverwalk, Citywest, Business Campus, Dublin, 24 Ireland; 4 Advanced Manufacturing Technology, GlaxoSmithKline, 709 Swedeland Rd, King of Prussia, Pennsylvania 19406-0939 USA

**Keywords:** 3D printing, paracetamol, sustained release, immediate release, personalised medicine, geometry

## Abstract

An extrusion-based 3D printer was used to fabricate paracetamol tablets with different geometries (mesh, ring and solid) from a single paste-based formulation formed from standard pharmaceutical ingredients. The tablets demonstrate that tunable drug release profiles can be achieved from this single formulation even with high drug loading (> 80% *w*/*w*). The tablets were evaluated for drug release using a USP dissolution testing type I apparatus. The tablets showed well-defined release profiles (from immediate to sustained release) controlled by their different geometries. The dissolution results showed dependency of drug release on the surface area/volume (SA/V) ratio and the SA of the different tablets. The tablets with larger SA/V ratios and SA had faster drug release. The 3D printed tablets were also evaluated for physical and mechanical properties including tablet dimension, drug content, weight variation and breaking force and were within acceptable range as defined by the international standards stated in the US Pharmacopoeia. X-ray powder diffraction, differential scanning calorimetry and attenuated total reflectance Fourier transform infrared spectroscopy were used to identify the physical form of the active and to assess possible drug-excipient interactions. These data again showed that the tablets meet USP requirement. These results clearly demonstrate the potential of 3D printing to create unique pharmaceutical manufacturing, and potentially clinical, opportunities. The ability to use a single unmodified formulation to achieve defined release profiles could allow, for example, relatively straightforward personalization of medicines for individuals with different metabolism rates for certain drugs and hence could offer significant development and clinical opportunities.

## INTRODUCTION

Personalised medicine is defined as a customization of health care to individual patients through linking diagnostics and treatments with genetic testing and emerging technologies such as proteomics and metabolomics analysis (1). The main advantages of this approach are to increase the effectiveness of the prescribed treatment regimen and to minimise their adverse effects such as those linked to overdosing of drugs with a narrow therapeutic index such as digoxin and anti-clotting agents (2). In the context of solid oral dosage forms, conventional large-scale tableting manufacturing methods are clearly unsuited to personalised medicine and, in addition, provide restrictions on the complexity achievable in the dosage form in terms of, for example, tablet geometry, drug dosage, distribution and combinations. 3D printing offers the potential for the manufacture of bespoke solid oral dosage forms. 3D printers also offer the possibility of reducing the number of manufacturing steps as currently used in traditional tablet production process, such as powder milling, wet granulation, dry granulation, tablet compression and coating and the potential for rapid formulation development with limited quantities of active ingredients as available in early drug development (3,4).

3D printing is hence a potentially significant platform that can produce viable solid dosage forms in complex geometries in a programmed, controlled manner and with accurate drug loading (5–8). Many believe that 3D printers could play an important role in the development of personalised unit dose medication for targeting the specific needs of individual patients and treatments (5,6,9). In envisaging how such an approach could be taken to the practical manufacture of dosage forms, it would clearly simplify matters greatly if the formulation (or ‘ink’ in 3D printer terms) could be kept as simple as possible, with little need for the use of multiple formulations that must be mixed precisely *in situ* within the 3D printer. Such a complex mixing approach would greatly complicate supply chains, increase quality control difficulties and subsequently raise regulatory barriers even higher than might be expected for such a new approach to manufacture. We propose, and demonstrate here, that the required need for personalization in terms of drug release profile can be achieved by the control of tablet geometry alone from a single formulation. Such an approach we propose would significantly increase the likelihood of 3D printing being adopted for the development and manufacture of personalised dosage forms.

Paracetamol is commercially available in many different dosage forms including tablets, capsules, suspensions, suppositories and intravenous solutions and is commonly used to treat mild to moderate pain caused by headaches, toothache, sprain or strains (4). Here, paracetamol was chosen as a well-known freely available drug suitable for a proof of concept study. The common paracetamol doses available range from 300 to 500 mg, although 1000 mg is also available in some regions. Therefore, customising of paracetamol effect/release (plasma peak levels) while prolonging its action by using different tablet geometries is potentially desirable (10). The effect of dosage form geometry on drug release for controlled release has been reported (10–12). Previously, work has also been done on 3D printing of paracetamol formulations primarily using fused deposition modelling (FDM) 3D printing (4,13–18). However, the high extrusion temperature used in FDM (≥ 120°C) narrows the potential active ingredient library to include only heat stable actives (4). Other possible 3D printing methods like stereolithography (SLA) and ink-jet printing currently use excipients that are not generally recognised as safe (GRAS) (13).

Different types of 3D printer are commercially available including the aforementioned FDM, inkjet, selective laser sintering (SLS) and SLA, and significant work has been done in the area of drug delivery using these approaches (7,12–14,19–23). Published research regarding 3D printing techniques to achieve controlled drug release include Sadia and co-workers, who created multi-channelled tablets using FDM for a Biopharmaceutics Classification System (BCS) class IV drug, hydrochlorothiazide (24). Also Yang et al. used FDM to print tablets with differing internal scaffold structures to control ibuprofen release (25). SLS has been used by Fina et al. to create orally disintegrating paracetamol tablets whose drug release depending upon the printing speed (17). We have also previously demonstrated the flexibility afforded by 3D extruding semi-solid formulations at ambient conditions using compendia grades available to form tablets to achieve controlled drug release (5,6,26). Whilst extrusion-based 3D printing avoids the heat stress associated with other techniques, it has some disadvantages including relatively low spatial resolution compared to other 3D printing approaches, and that it may not be suitable for water-sensitive materials (degradation unless solvent or binder other than water is used). In this research, the drying temperature was set at 80°C to accelerate the drying time of the printed tablets (4). However, lower temperatures in a range of 40–60°C can be employed, as is commonly used in drying oral solid dosage form but this leads to longer drying times. The aim of this work is to introduce extrusion-based 3D printer for the first time as a capable tool to print different geometries with meaningful drug loading that can be used to define drug release profiles.

## MATERIALS AND METHODS

### Materials

Paracetamol and polyvinylpyrrolidine (PVP K25) were supplied by Sigma–Aldrich (Gillingham, UK). Croscarmellose sodium (NaCCS) (Primellose®) was kindly supplied as a gift from DFE Pharma. Starch was kindly supplied by Colorcon®. Milli-Q water (resistivity 18.2 MΩ cm) was used for all formulations and solutions. All other reagents were of either HPLC or analytical grade.

### Methods

#### Design of Paracetamol Tablets

**Fig. 1:**
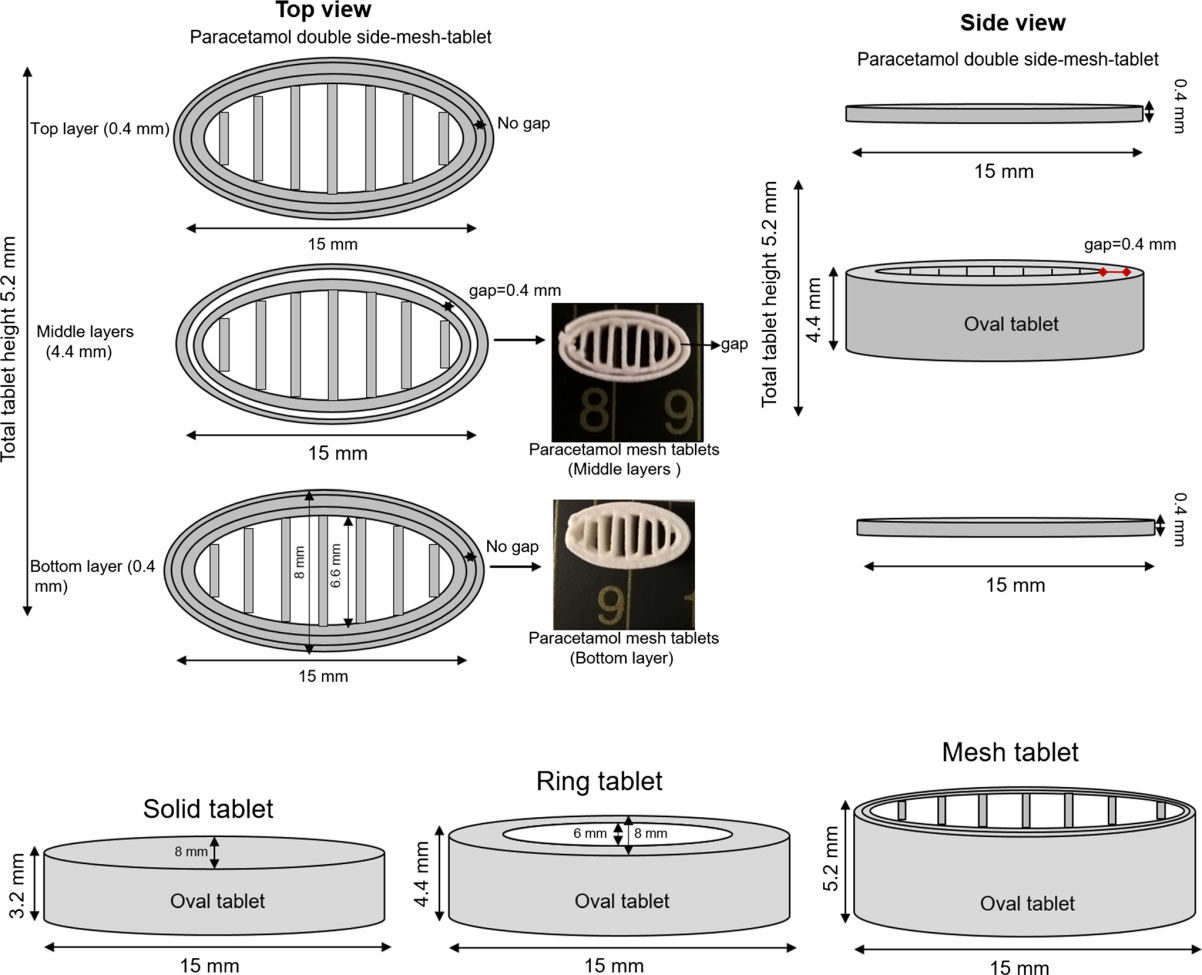
Schematic structural diagram of paracetamol 3D printed tablets with different geometric shapes; mesh, ring and solid tablets

A strategy of controlling the geometry to be generally oval shaped (easy to swallow) for the 3D printed tablets was chosen (Fig. 1). A normal solid tablet geometry was altered to also produce an oval ring and an oval tablet with an internal mesh or lattice-like structure. The mesh tablets which were printed in 13 layers in an external oval ring (formed from two or three printed ovals) and an internal cross-lattice mesh format. There was an internal gap of 0.4 mm between the two printed oval walls of layers 2–12 (Fig. 1), with the top (layer 13) and bottom (layer 1) layers having three oval walls printed around the mesh structure with no gap between them to ensure tablet integrity. The ring tablets were simply produced by printing oval walls of different dimensions until the ring-like structure was achieved. The outer dimensions of the designed oval tablets were 15 mm length × 8 mm width × 3.2 mm height for the solid tablets, 4.8 mm height for the ring tablets and 5.2 mm height for the mesh tablets. The geometry of the tablets was designed using a 3D drawing package (BioCAD, regenHU Villaz-St-Pierre, Switzerland) with the aim of keeping the tablet weight constant across the three geometries.

#### 3D Printing Process of Paracetamol Tablets

Twelve grams of ground paracetamol and the required excipient powders (starch, PVP K25 and NaCCS) were mixed using a mortar and pestle for 10 min. Milli-Q water (4.5 ml) (resistivity 18.2 MΩ cm) was added, and the powder was mixed to form a paste according to the formulae shown in Table I.

**Table I:** The Percentage Composition of Various Ingredients in Paracetamol Formulation Feedstock

Name of material	Function	Total formulae (mg)	Wt.% *w*/w (wet formulae)	Wt.% *w*/*w* (dry formulae)	Calc. drug weight (mg) (dry tablets)^*a*^
Paracetamol	API	810.42	58.94	81.04	249.42
PVP	Binder	100.00	7.27	10.00	30.78
Starch	Binder	83.33	6.06	8.33	25.64
CCS	Disintegrant	6.25	0.45	0.63	1.94
Water	Binder	375.00	27.27	–	–
Total	–	1375.00	100.00	100.00	307.78

*API* active pharmaceutical ingredient, *PVP* polyvinylpyrrolidone, *CCS* croscarmellose sodium

^*a*^Calculated from the average of the total paracetamol tablet weight (307.78 mg, *n* = 6)

#### Extrusion-Based 3D Printing Process

**Fig. 2:**
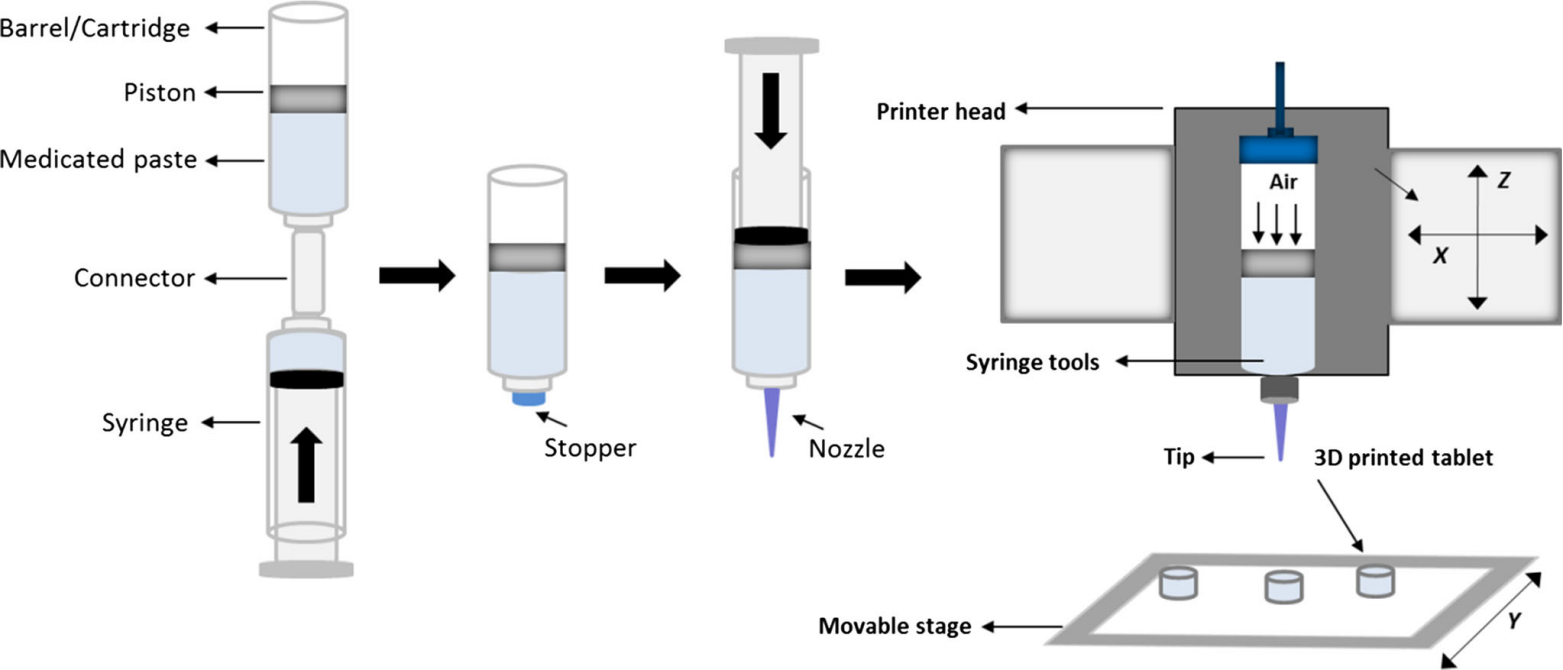
Schematic diagram of cartridge/barrel tool filling process

A plastic 20 cm^3^ syringe (Optimum® syringe barrels, Nordson EFD) was used to fill the paste into the syringe cartridge in the 3D printer (regenHU 3D). A stopper was fixed into Luer-Lock thread at the top end of the barrel after the filling process to avoid any unintentional leakage of paste from the cartridge showed in Fig. 2. Once ready for printing, the stopper was removed, and the required nozzle (Optimum® SmoothFlow™ tapered dispensing tips, 0.6 mm internal diameter (ID) Nordson EFD) installed. The filled cartridge was then installed into the printer head, and the paste was extruded layer by layer until the desired tablet dimension was reached (Fig. 2). The 3D printed tablets were left on a heated printing platform (80°C) overnight for complete drying. The tablets were stored in a sealed desiccator stored in a cool and dry location. The following printing parameters were used: tip diameter 0.6 mm, printing speed = 6 mm/s, printing pressure = 1.8 bar, number of printed layers = 13 for mesh tablets, 12 for ring tablets and 8 for solid tablets. The tablet outer dimensions were kept the same, but the geometries were varied using functions in BioCAD software. The tablet weights were kept constant within a measured range of 308.01 ± 4.52 mg by adjusting the printed tablet height.

#### Dissolution Studies

*In vitro* drug release studies of the paracetamol 3D printed tablets were performed using a USP Type I apparatus (rotation speed at 30 rpm, 900 ml phosphate buffer, pH 6.8 as the dissolution media at 37 ± 0.5°C). Samples (5.0 ml) were withdrawn at 5, 10, 15, 30, 60, 120, 240, 360, 480, 600 and 720 min. The samples were centrifuged and 0.5 ml from the supernatant was drawn and diluted to 10 ml using the dissolution medium. The samples were analysed with a UV–vis spectrophotometer (Cary® 50 UV-vis spectrophotometer) at a λ max of 243 nm. Drug dissolution studies were conducted in sextuplicate, and the average of percentage of cumulative drug release as a function of time was determined. Although the USP monograph specifications for paracetamol tablet dissolution testing state that the dissolution rotation should be 50 rpm, a speed of 30 rpm was chosen to ensure that the tablet disintegration occurred mainly due to the effect of disintegrants rather than effects caused by basket rotation.

### Characterisation Techniques

#### X-Ray Powder Diffraction (XRPD)

The XRPD patterns of pure paracetamol, excipients (PVP K25, NaCCS and starch) and paracetamol formulation powder (powder mixture after tablet ground into powder) were obtained at room temperature using an X’Pert PRO (PANalytical, Almelo, Netherlands) setup in reflection mode using Cu Kα1 (lambda = 1.54 Å) operating in Bragg–Brentano geometry. The generator voltage was set to 40 kV and the current to 40 mA, and the samples were scanned over 2*θ* range of 5° until 30° in a step size of 0.026^°^.

#### Attenuated Total Reflectance Fourier Transform Infrared Spectroscopy (ATR-FTIR)

Infrared spectra of pure paracetamol, excipients powders (PVP K25, NaCCS and starch) and paracetamol formulation powder (powder mixture after tablet ground into powder) were obtained using an ATR-FTIR (Agilent Cary 630 FTIR) spectrometer.

#### Differential Scanning Calorimetry (DSC)

The DSC measurements were performed on a TA Instruments’ DSC Q2000 coupled to Universal Analysis 2000 with a thermal analyser. DSC analysis on such drug-excipient mixtures were obtained by grinding paracetamol tablets and sieving the powders (< 150 μm). Accurately weighed samples of 3–5 mg were placed and sealed in aluminium pans. The scans were performed under nitrogen flow (50 mL/min) at a heating rate of 10°C/min from 35 to 200°C. An empty sealed aluminium pan was used as a reference.

### Physical Properties of Paracetamol Immediate Release 3D Printed Tablets

#### Dimension of Paracetamol 3D Printed Tablets

To confirm the tablet size reproducibility, six tablets from each geometry were measured using Vernier callipers and their average values calculated.

#### Weight Variation and Drug Content in the Final Tablet

Six paracetamol 3D printed tablets (from each geometry) were individually weighed and their average weight calculated. The individual tablet total weight deviation (%) was calculated. Paracetamol content in the final tablet was measured as follows: from each batch, 10 paracetamol tablets were weighed and crushed into powder. A quantity of paracetamol formulation powder equivalent to 0.25 g of paracetamol was weighed and transferred into a 1000 ml volumetric flask. Nine hundred millilitres of dissolution medium was added to the flask and placed on a stirrer for 4 h. Samples (5.0 ml) were withdrawn and centrifuged. From the supernatant, 0.5 ml was drawn and diluted to 10 ml using the dissolution medium. The samples were analysed with a UV–vis spectrophotometer (Cary® 50 UV-vis spectrophotometer) at a λ max of 243 nm. Content uniformity studies were conducted in triplicate, and the average of percentage of paracetamol content was determined.

#### Breaking Force

Six paracetamol 3D printed tablets (from each geometry) were randomly selected and tested for breaking force using a hardness tester (Hardness tester C50, I Holland Ltd., Holland). The breaking force values were recorded in N (Newton) units, and the tensile strength values were calculated using Eq. 1 (27,28). The tablet breaking force test was done parallel to the longest axis of the paracetamol tablets.

1 $$ {\sigma}_f=3 FL/2{bd}^2 $$

where *σ*_*f*_ is the tensile fracture strength of the tablet, *F* is the breaking force, *L* is the tablet length, *b* is the tablet width and *d* is the tablet thickness.

#### Friability

Ten paracetamol 3D printed tablets (from each geometry) were selected randomly, and the tablets were accurately weighed (initial weight). The tablets were placed in a friability tester and rotated at a constant speed of 25 rpm for a period of 4 min in Erweka friabilator. The tablets were cleaned of any loose dust and reweighed (final weight), and the weight loss % (friability) was calculated.

## RESULTS AND DISCUSSION

### Tablet Printing

**Fig. 3:**
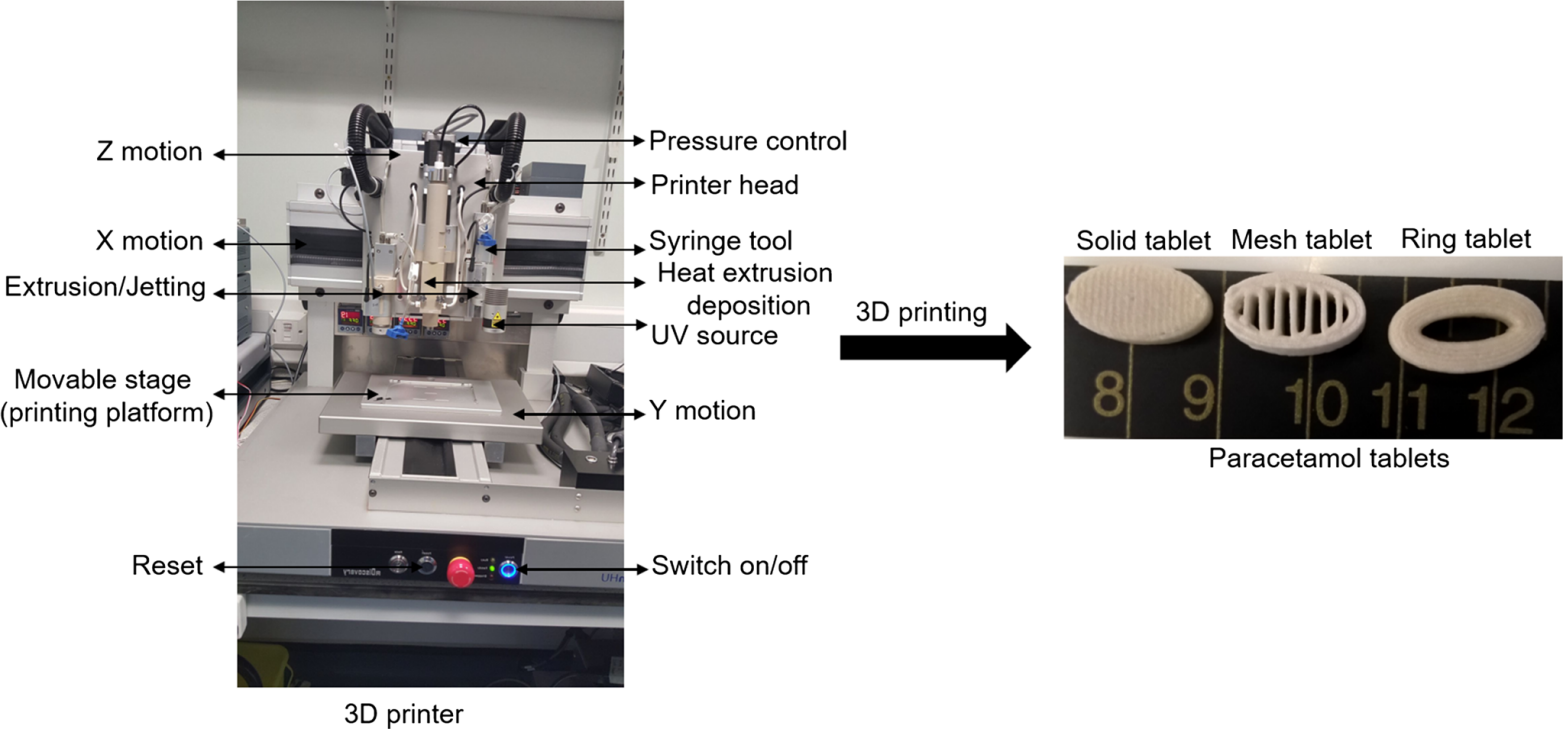
The regenHU 3D printer (left) and image of paracetamol tablets 15.35 mm length × 8.41 mm width × 3.44 mm height for solid tablets, 15.24 mm length × 8.41 mm width × 4.8 mm height for ring tablets, and 15.22 mm length × 8.48 mm width × 5.46 mm height for mesh tablets (average, *n* = 6) (right)

Batches of tablets were printed following the method outlined in Fig. 2. Examples of printed tablets are shown in Fig. 3.

### *In vitro* Drug Dissolution

**Fig. 4:**
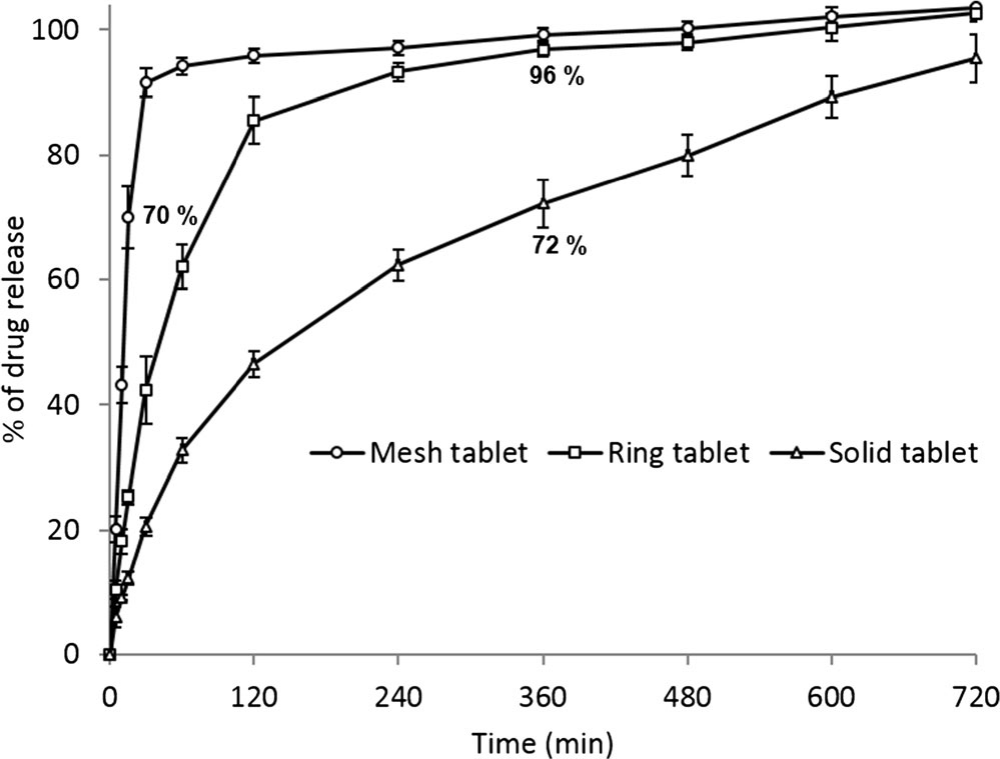
*In vitro* cumulative paracetamol release profiles from three different geometries; mesh, ring and solid paracetamol tablets, *n* = 6 (the printed tablets have different height but similar dimension and total weight and dose)

Dissolution data from the paracetamol tablets (Fig. 4) showed that the different tablet geometries with different height but similar dimension and total weight and dose (Tables IV and V) gave distinct release profiles. For the paracetamol mesh tablets, more than 70% of the drug was released within the first 15 min. In contrast, only 25 and 12% of the drug was released in the same period from the ring and the solid paracetamol tablets, respectively. This indicates that the tablet surface area showed an influence on drug release. Apart from surface area exposed to solution, the drug release is also impacted by the inclusion of the disintegrant, NaCCS, which rapidly absorbs water and swells leading to rapid disintegration. For the mesh tablets with the increased surface means that water absorption takes place more rapidly than for the ring and solid tablets (Fig. 4).

The drug release from the 3D printed tablets correlates with the SA/V ratios, the higher the SA/V ratio value, the faster the drug release (Table II). This trend has also been reported by other researches (10,11,29). Goyanes et al. showed the effects of SA/V ratios of different geometries on paracetamol release from tablets prepared by hot melt extrusion (HME) (11). Also in the same study, the authors showed that the drug release was independent of the surface area (11). Research done by Yi et al. demonstrated that the drug release from polylactic-co-glycolic acid/polycaprolactone/5-fluorouracil (PLGA/PCL/5-FU) patches was dependent on the changes of SA produced by geometric modifications (12). The authors then concluded that the tendency of slowing drug release corresponded to a decrease in the SA/V ratio (12). Furthermore, Gökçe et al. studied the influence of tablet SA/V ratio of two different geometries (cylinder and hexagonal) of the lipophilic matrix tablets of metronidazole prepared by Cutina HR (hydrogenated castor oil) (10). They found that the tablets with the highest release rates for both geometric shapes reflecting the highest surface area and the lowest SA/V ratio (10). Kyobula et al. showed that hot melt 3D inkjet printing can be used to manufacture complex and variable honeycomb geometry tablets for the controlled loading and release of the drug fenofibrate. In this case, the surface area and wettability of the tablet were shown to influence to the observed sustained drug release profiles (5). Hence, as can reasonably be expected, we can conclude that the tablet geometry and surface area generally have an effect on drug release behaviour and are parameters that can be manipulated to control drug release, even in formulations with additives such as a swellable disintegrant, as here. The higher the SA and SA/V ratio values, the faster the drug release is from the 3D printed tablets (Fig. 4 and Table II).

**Table II:** Paracetamol 3D Printed Tablet’s Dimensions for Different Geometries of Similar Total Weight and Increased Surface Area and SA/V Ratios

Geometry	Surface area (SA) (mm^2^)	Volume (V) (mm^2^)	SA/V ratio	Weight (mg)	Tablet dimension (mm)	Density (mg/mm^3^)
					*L* × *H* × *D*	
Mesh	897 ± 9.4	301 ± 3.9	2.976 ± 0.008	318 ± 11.1	15.2 ± 0.02 × 5.4 ± 0.05 × 8.5 ± 0.05	1.054 ± 0.023
Ring	449.94 ± 2.65	369.96 ± 3.25	1.216 ± 0.004	323.00 ± 1.70	15.3 ± 0.03 × 5.0 ± 0.06 × 8.5 ± 0.04	0.866 ± 0.005
Solid	330.94 ± 2.04	344.19 ± 5.19	0.962 ± 0.009	313.00 ± 9.20	15.4 ± 0.03 × 3.4 ± 0.06 × 8.4 ± 0.05	0.909 ± 0.013

*L* length, *H* height, *D* diameter

The demonstrated ability to use a single unmodified formulation to achieve defined release profiles presents opportunities to optimise or personalise medicines during formulation development and in clinical use. For example, relatively straightforward personalization of medicines would be possible for individuals with different metabolism rates due to their genetic makeup (26) for certain drugs and hence could address issues where people who metabolise drugs slowly may accumulate a toxic level of a drug in the body or in others who process a drug quickly and never have high enough drug concentrations to be effective.

### Drug Release Kinetics

To further understand the drug release mechanisms displayed by the different geometries, the modes of release of paracetamol over 12 h at a buffer pH 6.8 was modelled using zero-order, first-order, Higuchi and Korsmeyer–Peppas models (30,31). According to fitted *r*^2^ values, the mesh and ring tablets were best fitted by the first-order equation (*i.e.* log cumulative percentage of drug remaining is proportional to the time) (32) and the solid tablets were best fitted by the Higuchi model (*i.e.* cumulative percentage drug release *versus* square root of time) (32) with *r*^2^ values of 0.77, 0.97 and 0.99, respectively (Table III). The equation reveals *n* values (as in Eq. (2)) of 0.25 for mesh tablets, 0.44 for ring tablets and 0.56 for solid tablets.

2 $$ {M}_t/{M}_{\infty }={Kt}^n $$

**Table III:** Fitting Experimental Release Data, from the *In Vitro* Release of 3D Printed Paracetamol Tablets to Zero-order, First-order, Higuchi and Korsmeyer-Peppas Kinetic Equations at a Buffer Condition (pH 6.8–12 h)

Geometry	Zero order (*r*^2^)	First order (*r*^2^)	Higuchi (*r*^2^)	Korsmeyer-Peppas (*r*^2^)	*n* value
Mesh	0.38	0.77	0.53	0.64	0.25
Ring	0.67	0.96	0.84	0.91	0.44
Solid	0.91	0.98	0.99	0.98	0.56

where *M*_*t*_/*M*_∞_ is the fraction of drug released at time *t*, *K* is the release rate constant and the release exponent (32,33).

The above results suggest that the drug is released primarily by *Fickian diffusion* through a gel layer formed by the amylose in the added starch. Amylose is known to absorb water, swell and then form a gel layer (34). The drug release from the mesh tablets was faster than the drug release from the other geometries (ring and solid). This is, we propose, related to the larger surface area (mesh>ring>solid) and the more easily disrupted geometry of the mesh tablets where the chance to form a stable gel layer, and hence, retard drug release is inhibited. The disintegrants (the amylopectin (insoluble component found in the starch that can absorb water, swell and act as disintegrant) and NaCCS)) work to weaken and disrupt the formed gel layer in the mesh tablets. In case of ring and solid tablets, the geometry is more compact with a smaller surface area and less exposure to the dissolution medium than mesh tablets, so the disintegration rate is reduced, and there is an increased time to form a gel layer and hence retardation of drug release (solid>ring>mesh).

### XRPD

**Fig. 5:**
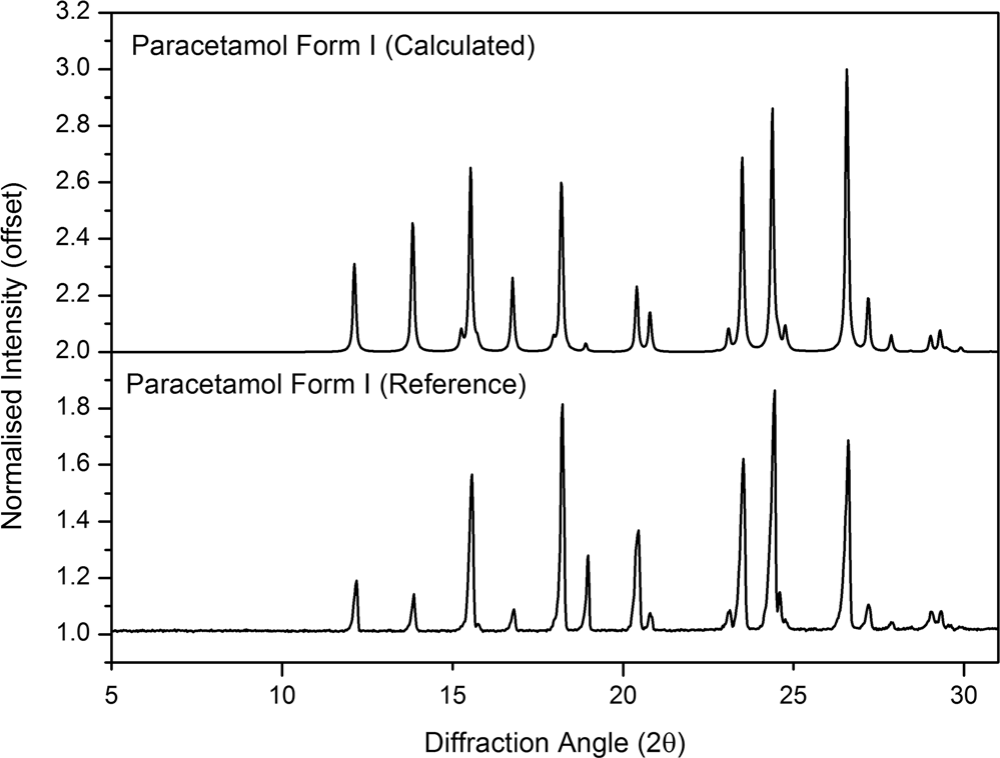
XRPD patterns of the calculated (top) and reference (measured) paracetamol

**Fig. 6:**
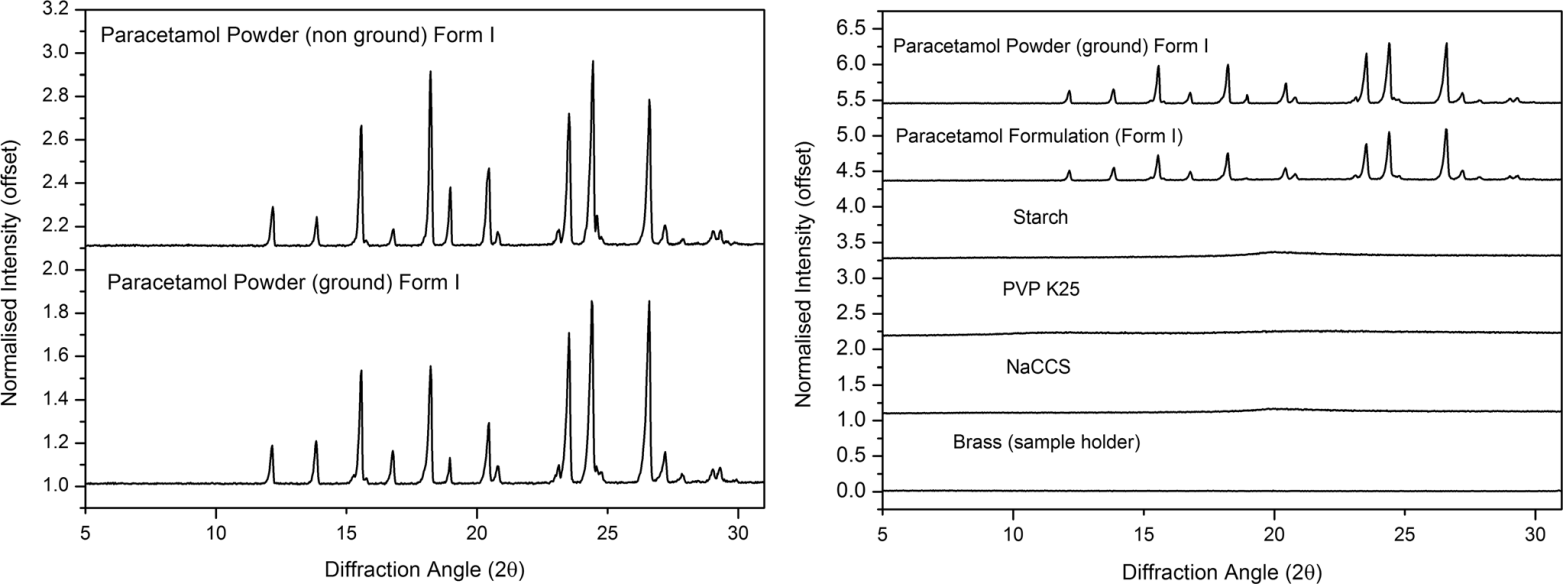
XRPD patterns of paracetamol powder (non-ground and ground form I) (left), paracetamol powder (ground form I), paracetamol formulation, starch, PVP K25, NaCCS and brass (sample holder) (right)

XRPD of the pure paracetamol, excipients (PVP K25, NaCCS and starch) and paracetamol formulation powder (powder mixture after tablet ground into powder) was done to investigate any potential changes in physical form of the active on printing (Figs. 5 and 6). The Bragg peaks observed from the pure paracetamol (as received) match the Bragg peaks of paracetamol (calculated) reported in the Cambridge Structural Database (CSD) (Fig. 5).

The results in Fig. 6 show that the paracetamol (non-ground and ground powder) exhibited multiple sharp Bragg peaks in their XRPD patterns related to their crystalline nature. The post-printing XRPD data show the same Bragg peaks for the paracetamol. There was, therefore, no evidence of a change in physical form (form I) for the paracetamol in this formulation fabricated using extrusion-based 3D printing. We believe that a portion of the paracetamol powder could have dissolved after addition a significant quantity of water (4.5 ml) into total paracetamol dry formulae (12 g) (paracetamol solubility 12.78 g/l /20°C) (34) as the whole mixture formed a paste; however, this must have recrystallised back into form I if this had occurred. The XRPD data from Fig. 6 also did not show evidence of incompatibility between the active and the chosen additives (PVP K25 (10% *w*/*w*), starch (8.33% *w*/*w*) and NaCCS (0.63% *w*/*w*)) in the 3D printed tablets.

### ATR-FTIR

**Fig. 7:**
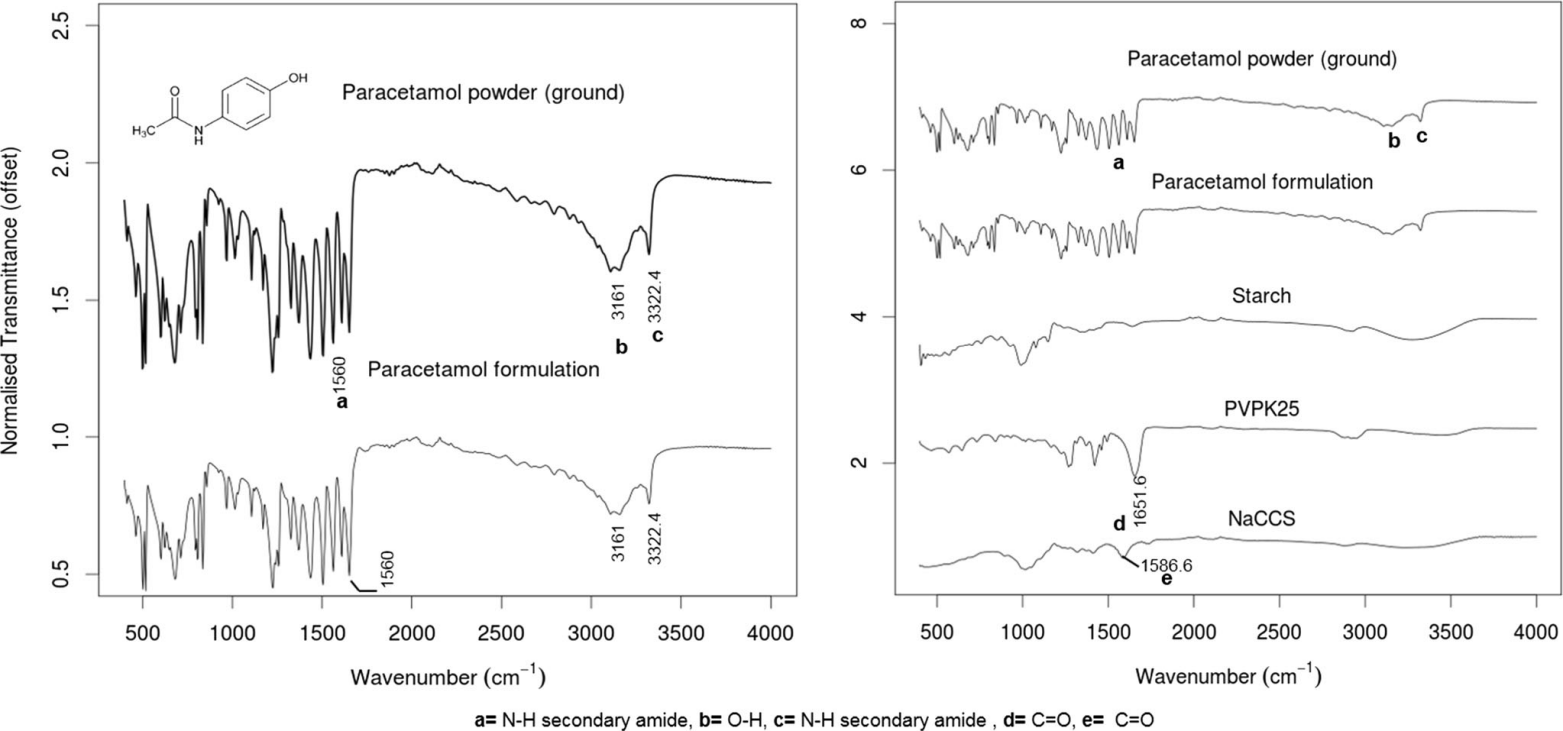
FTIR spectra of paracetamol powder (ground form I) and paracetamol formulation (left), starch, PVP K25, NaCCS (right)

Infrared spectral data show that the characteristic peak positions remained unchanged from the paracetamol powder to the formulation, indicating that there were no detectable interactions between paracetamol (81% *w*/*w*) and the chosen excipients (PVP K25 (10% *w*/*w*), starch (8.33% *w*/*w*) and NaCCS (0.63% *w*/*w*)) in the tablets (Fig. 7).

### DSC

**Fig. 8:**
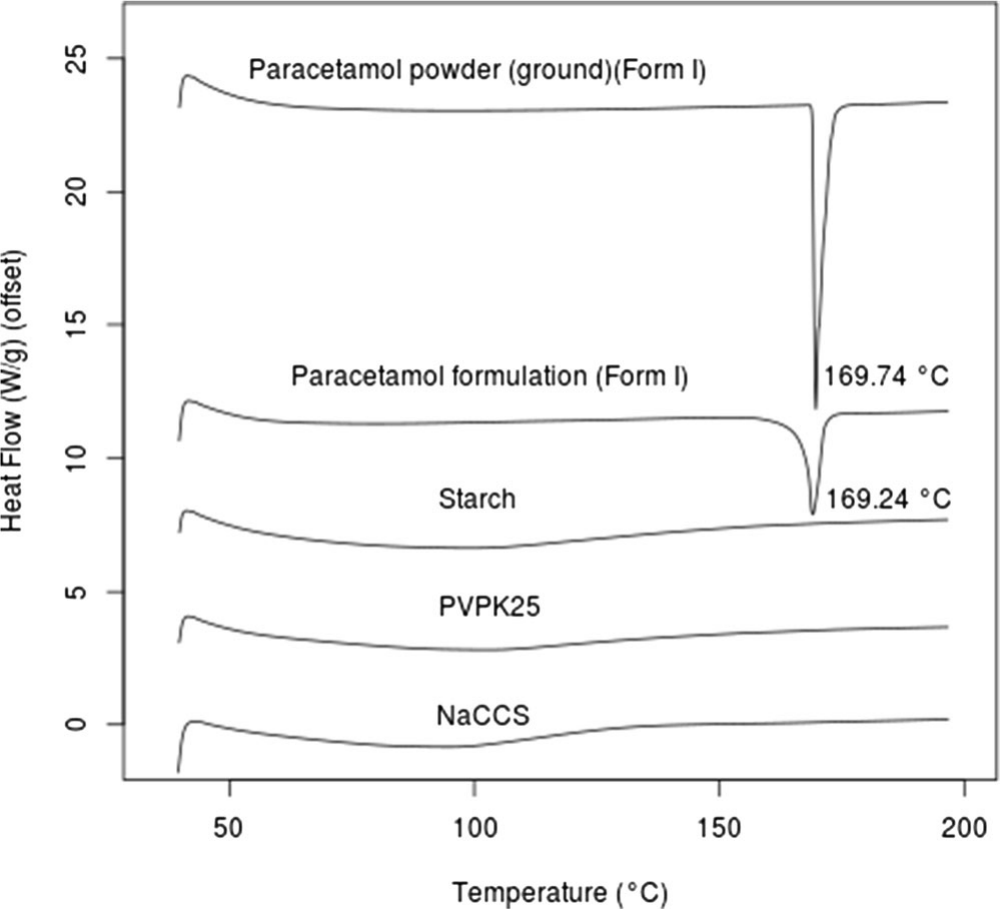
DSC thermograms of pure paracetamol, paracetamol formulation, starch, PVP K25 and NaCCS

DSC analysis was performed to explore potential incompatibility between the active and added excipients and the stability of drug crystallinity after the 3D printing process (grinding, mixing, paste formulation and drying process on a hot plate heated at 80°C). The DSC data from Fig. 8 shows that the pure powder of paracetamol melts at 169.7°C confirming the presence of form I (4,35,36) while the pure powder of PVP K25 shows a glass transition (T_g_) around 155°C (4,37). The same figure also shows clear evidence of an endothermal event (melting point) at 169.24°C from the printed paracetamol formulation, indicating that the active is still in a crystalline form, specifically form I. From the above results and discussions, we found that DSC thermogram of paracetamol formulation powder after grinding, blending, printing and post-printing processes with the excipients; starch, PVP K25 and NaCCS did not show significant changes in peak placement apart from the peak depression and reduction caused by the presence of the polymer in the formulation in comparison to the peak obtained from the pure paracetamol powder and again suggesting compatibility of the excipients.

### Physical Properties

The 3D printed tablets were evaluated for weight variation, content uniformity, breaking force, friability and tablet dimensions.

#### Tablet’s Shape and Dimension

Table IV confirms that the tablet dimensions were reproducible and comparable with the designed tablet’s size and dimension and with the tablet size reported in the literature prepared by conventional tableting press machines (38–40).

**Table IV:** Individual Paracetamol 3D Printed Tablet’s Dimensions and Their Average, Median, Maximum, Minimum Dimension and Standard Deviation

Tablet no.	Mesh tablets (mm)			Ring tablets (mm)			Solid tablets (mm)		
	Length	Height	Width	Length	Height	Width	Length	Height	Width
1	15.24	5.42	8.50	15.01	5.13	8.30	15.39	3.50	8.35
2	15.21	5.51	8.47	15.33	5.08	8.47	15.40	3.46	8.47
3	15.20	5.40	8.38	15.38	4.94	8.50	15.34	3.36	8.46
4	15.22	5.46	8.51	15.30	5.07	8.40	15.36	3.52	8.33
5	15.26	5.45	8.53	15.26	5.09	8.42	15.26	3.38	8.42
6	15.19	5.49	8.47	15.16	5.06	8.38	15.37	3.42	8.45
Average	15.22	5.46	8.48	15.24	5.06	8.41	15.35	3.44	8.41
Median	15.22	5.46	8.49	15.28	5.08	8.41	15.37	3.44	8.44
Maximum	15.26	5.51	8.53	15.38	5.13	8.50	15.40	3.52	8.47
Minimum	15.19	5.40	8.38	15.01	4.94	8.30	15.26	3.36	8.33
SD	0.03	0.04	0.05	0.13	0.06	0.07	0.05	0.06	0.06

#### Weight Variation

The paracetamol 3D printed tablets showed an acceptable percentage weight variation (Table V) and, therefore, comply with the USP specification for uncoated tablets (± 7.5% for average weight of tablets 130–324 mg) (41,42). The paracetamol content in the final tablets was also assessed and found to be 103.2 ± 1.1% for the mesh tablets, 104.0 ± 1.1% for the ring tablets and 103.1 ± 1.5% for the solid tablets.

**Table V:** Individual Paracetamol 3D Printed Tablets Weight, Calculated Paracetamol Dose/Tablet, Percentage Deviation, and Their Average, Median, Maximum, Minimum Weight and Standard deviation

Tablet no.	Ring tablet			Mesh tablet			Solid tablet		
	Tablet weight (mg)	Calc. para. dose/tablet	Deviation %	Tablet weight (mg)	Calc. para. dose/tablet	Deviation %	Tablet weight (mg)	Calc. para. dose/tablet	Deviation %
1	312.90	253.57	0.78	308.80	250.25	0.33	307.10	248.87	0.44
2	318.80	258.36	2.68	300.70	243.69	− 2.30	302.20	244.90	− 1.16
3	309.90	251.14	− 0.19	311.90	252.76	1.34	301.30	244.17	− 1.46
4	307.70	249.36	− 0.90	312.60	253.33	1.57	306.40	248.31	0.21
5	310.80	251.87	0.10	306.00	247.98	− 0.58	306.60	248.47	0.28
6	302.80	245.39	− 2.47	306.70	248.55	− 0.35	310.90	251.95	1.68
Average	310.48	251.62	0.00	307.78	249.43	0.00	305.75	247.78	0.00
Median	310.35	251.51	− 0.04	307.75	249.40	− 0.01	306.50	248.39	0.25
Maximum	318.80	258.36	2.68	312.60	253.33	1.57	310.90	251.95	1.68
Minimum	302.80	245.39	− 2.47	300.70	243.69	− 2.30	301.30	244.17	− 1.46
SD	5.33	4.32	1.72	4.38	3.55	1.42	3.52	2.85	1.15

#### Breaking Force

Table VI shows the 3D printed tablets breaking forces (kg and N) and the tensile fracture strength. Tensile fracture strength of the paracetamol flat-faced oval tablets was calculated (28). In a conventional tableting press, compression forces can be used to control the physical properties of the final tablet, where a breaking force value of 4 kg is the minimum satisfactory measurement (26,43). Measured breaking force measurements were within the accepted range of 8.69–9.56 kg for the solid tablets but failed to reach the minimum satisfactory value for the mesh and ring tablets (Table VI). It is clear that as compression force is not part of 3D printing process that the same opportunity to manipulate tablet hardness in this way does not exist and rather the formulation composition, solidification/drying process and the type of printer employed are critical factors. Clearly, further work beyond the scope of this paper is required in this area; however, from a subjective and qualitative assessment, the ring and mesh paracetamol 3D printed tablets appear to be quite robust and are able to tolerate a reasonable amount of rough handling. For example, they could be dropped onto a hard surface from a height of around 15 cm without observable damage. In addition, such tablets could be considered for manufacture close to the patient where traditional wear factors such as chipping, capping and abrasion which normally occurred during manufacturing, packaging and shipping processes are not relevant.

**Table VI:** Individual Paracetamol 3D Printed Tablet’s Breaking Force (kg and N), Tensile Fracture Strength (MPa), and Their Average, Median, Maximum, Minimum Hardness and Standard Deviation

Tablet no.	Mesh tablets			Ring tablets			Solid tablets		
	Breaking force (kg)	Breaking force (N)	Tensile strength (Mpa)	Breaking force (kg)	Breaking force (N)	Tensile strength (Mpa)	Breaking force (kg)	Breaking force (N)	Tensile strength (MPa)
1	2.56	25.11	2.30	2.50	24.53	2.53	8.69	85.25	19.24
2	2.40	23.54	2.09	2.80	27.47	2.89	9.15	89.76	20.45
3	2.70	26.49	2.47	2.50	24.53	2.73	8.71	85.45	20.59
4	2.39	23.45	2.11	2.26	22.17	2.36	9.04	88.68	19.80
5	2.60	25.51	2.30	2.57	25.21	2.65	8.93	87.60	20.85
6	2.44	23.94	2.14	2.49	24.43	2.59	9.56	93.78	21.88
Average	2.52	24.67	2.24	2.52	24.72	2.63	9.01	88.42	20.47
Median	2.50	24.53	2.22	2.50	24.53	2.62	8.99	88.14	20.52
Maximum	2.70	26.49	2.47	2.80	27.47	2.89	9.56	93.78	21.88
Minimum	2.39	23.45	2.09	2.26	22.17	2.36	8.69	85.25	19.24
SD±	0.12	1.23	0.15	0.17	1.70	0.18	0.32	3.17	0.91

#### Friability

This is a USP test used to determine a tablet resistance to abrasion, capping and chipping occurred during manufacturing, packaging and shipping processes. All paracetamol 3D printed tablets of different geometries showed a satisfactory percentage of weight loss ≤ 1% of the tablet weight (Table VII) and, therefore, the tablets meet USP specifications (44).

**Table VII:** Friability of Different Paracetamol 3D Printed Geometries; Mesh, Ring and Solid Tablets

Tablet	Friability (%)	Comment
Mesh	0.65	Pass
Ring	0.62	Pass
Solid	0.59	Pass

## CONCLUSIONS

Extrusion-based 3D printing of different paracetamol tablet geometries with a high drug loading (81% *w*/*w*) was successfully demonstrated. The mesh-geometry 3D printed tablets released more than 70% of the active within 15 min achieving immediate release mesh shaped tablets. In contrast, only 25 and 12% of the drug was released in the same period from the ring and the solid paracetamol tablets, respectively, effectively demonstrating sustained release. Drug release from the tablets showed a clear dependency on the SA/V ratio. XRPD, FTIR and DSC data show that the paracetamol form was unaffected by the printing and that there were no detectable interactions between the paracetamol and the chosen excipients (starch, PVP K25 and NaCCS). The 3D printed paracetamol tablets were also evaluated for weight variation, drug content in the final tablets, hardness, friability and tablet dimensions and were within acceptable range as defined by the international standards stated in the USP. This work again validates that the extrusion-based 3D printing process is capable of producing viable tablets from materials having compendia grades available for pharmaceutical applications. More importantly this work demonstrates for the first time the application of extrusion-based printing for tailoring of drug release from a single formulation through control of only tablet geometry the first. We believe this is a significant step forward in the potential wider take up of 3D printing for the manufacture of medicines, particular in the areas of clinical development and personalised medicines. With this principal demonstrated, it becomes possible to envisage control of drug release and dose (through dosage form size) on an individual basis using a 3D printer, without the need for forming complex mixtures from different formulation ‘cartridges’. This would greatly simplify potential supply chains of formulation inks and the quality control of the printed product.
